# The association between osteopontin gene polymorphisms, osteopontin expression and sarcoidosis

**DOI:** 10.1371/journal.pone.0171945

**Published:** 2017-03-02

**Authors:** Hadas Lavi, Miri Assayag, Assaf Schwartz, Nissim Arish, Zvi G. Fridlender, Neville Berkman

**Affiliations:** Institute of Pulmonary Medicine, Hebrew University Hadassah Medical Center, Jerusalem, Israel; University of Birmingham, UNITED KINGDOM

## Abstract

**Background:**

Sarcoidosis is a systemic inflammatory disease of unknown etiology. Osteopontin (SPP1, OPN) is an extra cellular matrix glycoprotein and cytokine with a known role in granuloma formation and in autoimmune and inflammatory diseases.

**Objective:**

To determine whether plasma OPN levels are elevated in patients with sarcoidosis and compare the frequency of four single nucleotide polymorphism (SNPs) variants in the OPN gene in sarcoidosis patients compared to healthy controls.

**Methods:**

Demographic and clinical information, radiological studies and pulmonary function tests were evaluated in 113 patients with sarcoidosis and in 79 healthy controls. Blood samples were analyzed for SNPs of the OPN gene and for plasma OPN and CRP levels. Association between clinical features of disease and OPN levels as well as SNP frequencies was determined.

**Results:**

Plasma OPN levels were higher in sarcoidosis patients than in healthy subjects, (median: 217 vs 122ng/ml, p<0.001). Area under the curve for receiver operator curves (ROC) was 0.798 (0.686–0.909 95% CI.) No differences were observed between sarcoidosis patients and controls in the frequency of any of the SNPs evaluated. Presence of lung parenchymal involvement was associated with SNP distribution at rs1126772 (p = 0.02). We found no correlation between SNPs distribution and plasma OPN levels.

**Conclusions:**

Osteopontin protein levels are elevated in sarcoidosis. We found no evidence for an association between SNPs on the osteopontin gene and plasma OPN levels or the presence of sarcoidosis, however, an association between genotype and several phenotypic clinical parameters of disease was observed.

## Introduction

Sarcoidosis is a systemic inflammatory disease of unknown etiology, characterised by non-caseating granuloma formation in various organs, with several recognized genetic and environmental risk factors. The prevalence of sarcoidosis varies from 4.7–64 per 100,000, with an estimated annual incidence of 1.0–35.5 in 100,000 [1]. In a study conducted in northern Israel an annual incidence of 2 in 100,000 was found [2], with a ten-fold increase in disease incidence from 1980 to 1996.

Genetic susceptibility to sarcoidosis has been found to be independently related to both HLA Class I and HLA Class II groups such as HLA-DRB1 [3], HLA-DR5 [4]. HLA groups are not only related to susceptibility for sarcoidosis, but also to its clinical course. Extra pulmonary manifestations of sarcoidosis, and specifically Löfgren's syndrome, defined by a triad of erythema nodosum (EN), arthralgia and hilar lymphadenopathy, have been associated with the human leukocyte antigen (HLA) group DRB1 in European population [5]. HLA class II alleles are associated with several phenotypes: DRB1*0401 with ocular involvement, DRB3 with bone marrow involvement in blacks, and DPB1*0101 with hypercalcemia in whites [3]. Due to linkage disequilibrium between HLA groups, it is sometimes hard to determine which is the involved genetic predisposing factor, as in the case of HLA-DRB1 and HLA-DQB1, as both were correlate to sarcoidosis, and to one another [5]. Genetic susceptibility to sarcoidosis has also been found to be related to specific genes such as Butyrophilin-like protein 2 (BTNL2), which Belongs to the immunoglobulin superfamily [6], Annexin A11 (ANAXA11), which gives rise to auto-antibodies in several inflammatory diseases, including rheumatoid arthritis, systemic lupus erythematosus and Sjögren syndrome [7], Solute carrier family 11 (Proton-coupled divalent metal ion transporter), member 1 (SLC11A1), which is associated with risk of intracellular pathogens such as tuberculosis, but also with autoimmune diseases such as rheumatoid arthritis, crohn's disease, type 1 diabetes, and primary biliary cirrhosis [8]; and to Interferon alpha (IFNA) genes polymorphisms [9], known for its involvement in Th1 diseases. TNF-β and TNF-α polymorphisms are associated with susceptibility to sarcoidosis in certain populations [10], with TNF being a key regulator of the inflammatory response. Sarcoidosis is often associated with elevated serum Angiotensin-converting enzyme (ACE) levels. An ACE *Insertion/Deletion* polymorphism has been tested for association with the risk of sarcoidosis. Published results are unequivocal, however, it was found that genotyping for this I/D polymorphism improves the diagnostic value of serum ACE levels measurements [11,12].

Certain phenotypes in sarcoidosis have a genetic component. Early-onset sarcoidosis, together with familial Blau syndrome is associated with mutations of Nucleotide-binding oligomerization domain-containing protein 2 (NOD2), also known as caspase recruitment domain-containing protein 15 (CARD15), that cause constitutive NF-kappa-B activation. NOD2 mutations are related to Crohn's disease as well [13]. Sarcoidosis-related-uveitis is associated with a certain SNP of Heat Shock Protein 70/Hom. Moreover, the haplotype of *HSP70* can be used to discriminate it from idiopathic uveitis [14]. Several studies have described d a connection between the presence of Mycobacterium tuberculosis heat-shock proteins in sarcoidosis patients and polymorphisms of genes encoding for FC receptor γ. This suggests that reduced clearance of TB immune complex may be relevant in the pathogenesis of sarcoidosis [15]. Familial clusters have been described, but are relatively uncommon. An association between sarcoidosis and CD14, an LPS co-receptor, has been previously reported by us [16].

Osteopontin (OPN), also known as secreted phosphoprotein 1 (SPP1) and early T lymphocyte activation 1 (ETA1), is a secreted phosphoprotein, part of the small integrin-binding ligand N-linked glycoprotein (SIBLING) family [17]. It was first identified as a bone matrix protein, subsequently identified as a cytokine, and is involved in carcinogenesis, tissue formation and the immune response [18,19]. CD44 variants and several integrins serve as receptors for OPN. It interacts with most of these integrins through a central arginine-glycine-aspartate (RGD) domain, and has a heparin binding site and a thrombin cleavage site [20,21]. OPN skews the immune response towards Th1, interacting with αVβ3 integrin inducing pro-inflammatory IL-12 and suppressing IL-10 production [22]. Elevated levels of OPN are found in plasma or serum and tissue specific fluids of patients suffering from several granulomatous disorders including tuberculosis and silicosis [23,24], and from Th-1 related disorders such as inflammatory bowel disease [25,26], systemic lupus erythematosus [27], certain types of multiple sclerosis [28,29] and rheumatoid arthritis [30]. Elevated expression of OPN has been described in sarcoidosis granulomas and in the plasma from patients with sarcoidosis [31,32].

The OPN gene resides on the long arm of chromosome 4. Crosby et al [33] showed that the SPP1 gene comprises 7 exons, 6 of which contain coding sequences. According to the Single Nucleotide Polymorphism Database (dbSNP), of OPN (SPP1) known SNPs, 404 are Single Nucleotide Variants (SNV), as opposed to deletions, insertions, copy number variations or microsatellites. Some of them are related to several autoimmune and granulomatous disorders and pulmonary diseases [34–39]. A possible relationship between Sarcoidosis and OPN gene has been suggested previously in the Slovenian population, with certain haplotype serving as a protective factor for the disease [40]. The current study focuses on four SNPs: rs1126616 is a coding synonymous and exonic splicing enhancer variant; rs1126772 and rs9138 are 3-prime untranslated region (UTR) variants and rs4754 is a coding synonymous variant. We measured plasma OPN levels as well as the frequency of 4 polymorphic sites for the OPN gene in a cohort of 113 sarcoidosis patients and compared these to a cohort of 79 healthy controls.

## Methods

### Study population

Patients: Patients were recruited from two large outpatient pulmonary clinics in Jerusalem, Israel between January 2011 and September 2015. All participants signed an Informed Consent Form and the study was approved by the Hadassah Medical Center Ethics Committee. Charts of patients with biopsy findings compatible with sarcoidosis as well as a compatible clinical picture as determined by symptoms, laboratory abnormalities and/or imaging and without another identified cause of granulomatous disease were included in the cohort.

Demographic and clinical information including radiological and pulmonary function tests was gathered from patient questionnaires, hospital medical records, and outpatient medical records obtained from treating physicians’ records. All data and radiological studies were reevaluated by the pulmonary physicians and radiologists at the Hadassah Medical Center. Assessment of disease extent, severity, duration of symptoms prior to diagnosis, and extent and nature of organ involvement in the disease was evaluated. For this study, we used lung function tests (LFT) that were performed prior to and as close to blood drawing as was available.

Controls: A cohort of healthy subjects with no significant medical background was recruited as a control group.

### Sample collection

A single sample of 15ml venous blood into commercially available EDTA-treated tubes for DNA analysis and plasma analysis of osteopontin and CRP levels was obtained from patients and control subjects.

### Evaluation of osteopontin (SPP1/OPN) levels

Plasma was separated from the blood samples and frozen at (-20)°C. OPN and CRP levels in the plasma were measured using the commercially available kits (R&D Systems Inc., MN, USA). The detection range for the OPN ELISA is 62.5pg-4000pg/ml, the detection range for CRP ELISA is 15.6-1000pg/ml. Normal range for CRP 1–3 mg/L. The samples were diluted when needed.

### Genotype analysis

Genomic DNA was extracted from anticoagulated whole blood collected in EDTA from patients and controls. Isolation of DNA was done by phenol chloroform extraction [41] or using a salting out protocol, followed by alcohol precipitation [42].

Four different single nucleotide polymorphisms in the OPN gene were evaluated: rs4754, rs1126616, rs9138 and rs1126772 using commercially available primer/probe pairs: TaqMan^®^ Predesigned SNP Genotyping Assays containing sequence-specific forward and reverse primers, and two MGB probes: VIC^®^-labeled probe and FAM^™^-labeled probe, together with TaqMan^®^ Genotyping Master Mix according to protocol provided by the manufacturer (Applied Biosystems^®^). SNP genotyping was performed using StepOnePlus^™^ Real-Time PCR System. The results were obtained by TaqMan^®^ Genotyper^™^ Software [43].

### Statistical analysis

Data was analyzed using SPSS version 20.0 Software (SPSS Inc., Chicago, IL). P-value of 0.05 was considered statistically significant.

## Results

### Study population and patient characteristics

A total of 113 sarcoidosis patients and 79 control subjects were recruited for this study between January 2011 and September 2015. Tables 1 and 2 present clinical and demographic data of the patients group. The patients group consisted of 18% of Arab origin and 80% of Jewish origin. A family history of sarcoidosis was present in 5% of cases.

**Table 1:** Demographic characteristics of patients with sarcoidosis.

		% of patients (n)
Age at diagnosis (years)	All	100 (109)
	20–30	3 (3)
	31–40	10 (11)
	41–50	27 (29)
	51–60	35 (38)
	61–70	17 (19)
	>70	8 (9)
Gender	All	100 (112)
	Male	37 (41)
	Female	63 (71)
Smoking status	All	100 (106)
	Active/past smoker	23 (24)
	Never smoker	77 (82)

**Table 2:** Clinical characteristics of sarcoidosis patients.

		% of patients (n)
Pathological involvement	All	100 (113)
	Lung	73 (83)
	Thoracic lymph nodes	10 (11)
	Extra thoracic lymph nodes	7 (8)
	Liver	3 (3)
	Bone marrow	1 (1)
	Other	6 (7)
Scadding chest radiographic class	All	100 (104)
	0	6 (6)
	1	17 (18)
	2	62 (64)
	3	10 (10)
	4	6 (6)
Radiology findings (pulmonary CT scan)	All	100 (102)
	Symmetric Hilar lymphadenopathy^1^	42(13/31)
	Hilar lymphadenopathy^1^	65 (20/31)
	Mediastinal lymphadenopathy^1^	77 (24/31)
	Interstitial non-nodular	52(53)
	Nodular	50 (51)
	Other imaging	5 (5)
Extent of disease)objective and/or subjective) or organ involvement	All	100 (110)
	Lung	88 (97)
	Thoracic lymph nodes	77 (85)
	Hilar lymphadenopathy^1^	68 (21/31)
	Parenchymal disease	40 (44)
	Lymphadenopathy^1^^,^^2^	84 (26/31)
	Extra thoracic lymph nodes	19 (21)
	Skin	11 (12)
	Eyes	14 (15)
	Joints	25 (28)
	Hypercalcemia	5 (6)
	Hypercalciuria^1^	8 (2/26)
	Neurosarcoid	5 (5)
	Parotid/salivary gland^1^	10 (3/31)
	Spleen^1^	13 (4/31)
	Liver	5 (5)
	Heart	3 (3)
	Other	5 (6)
Presenting symptoms	All	100 (34)
	Dyspnoea	38 (13)
	Cough	44 (15)
	Fever/weight Loss	26 (9)
	Eyes	15 (5)
	Joints	35 (12)
	Rash/skin manifestation	12(4)
	Asymptomatic	12(4)
	Fatigue	12(4)
	Other	41 (14)
Symptoms	All	100 (107)
	Asymptomatic	14 (15)
	Cough	59 (63)
	Dyspnoea	56 (60)
	Fever/weight loss	17(18)
	Visual disturbances	7 (7)
	Arthralgia	30 (32)
	Rash	12 (13)
	Other	21 (23)
Duration of symptoms prior to diagnosis	All	100 (73)
	<1 month	14 (10)
	1–3 months	36 (26)
	4–6 months	14 (10)
	7–12 months	15(11)
	>12 months	22 (16)
Drugs at sampling	All	100 (32)
	Oral/IV Steroids	19 (6)
	ICS±LABA	3 (1)
	MTX	0 (0)
	Other	9 (3)
Treatment^3^	All	100 (105)
	Oral steroids	44 (46)
	ICS^1^	19 (6/32)
	MTX	7 (7)
	Other drugs	24 (25)
Abnormal PFT^4^	Present	100 (107)
		**median (IQR)**
Pulmonary function tests% of predicted	FVC	90 (77.8–103)
	FEV1	87.5(78–99.5)
	FEV1/FVC	84(77–91)
	VC	95(81.5–105.3)
	FRC(TGV)	97(81.5–114.5)
	TLC	95(83–105.5)
	RV	107(94–128)
	DLCO	84(72.5–95)
Bronchoalveolar lavage % cells	All	100 (24)
	BAL %lymphocyte	10.5 (0.5–31.5)
	BAL %macrophage	81.5(63.8–92.8)

Definition of abbreviation:

^1^pulmonary or systemic lymphadenopathy

^2^Data available for limited number of patients

^3^Inhaled corticosteroid

^4^Present or past treatment

^5^Defined as TLC<80 or FEV/FVC<71 or DLCO<80 or as defined by a physician.

**Definition of abbreviations**: **IQR**- interquartile range **LN**-lymph nodes **PFT**- pulmonary function test, **FVC**- Forced vital capacity **FEV1**- Forced expiratory volume in 1 second **FEV1/FVC**- Forced vital capacity divided by forced expiratory volume in 1 second **VC-** vital capacity **FRC (TGV (**- Functional Residual Capacity, (Thoracic gas volume) **TLC**- total lung capacity RV- residual volume **DLCO**- diffusing capacity for carbon monoxide, **ICS**- inhaled corticosteroid **MTX**-methotrexate **LABA**- long acting beta agonists **ICS±ABA**- inhaled corticosteroid, with or without long acting beta agonists.

### Osteopontin levels in sarcoidosis patients

**Fig 1:**
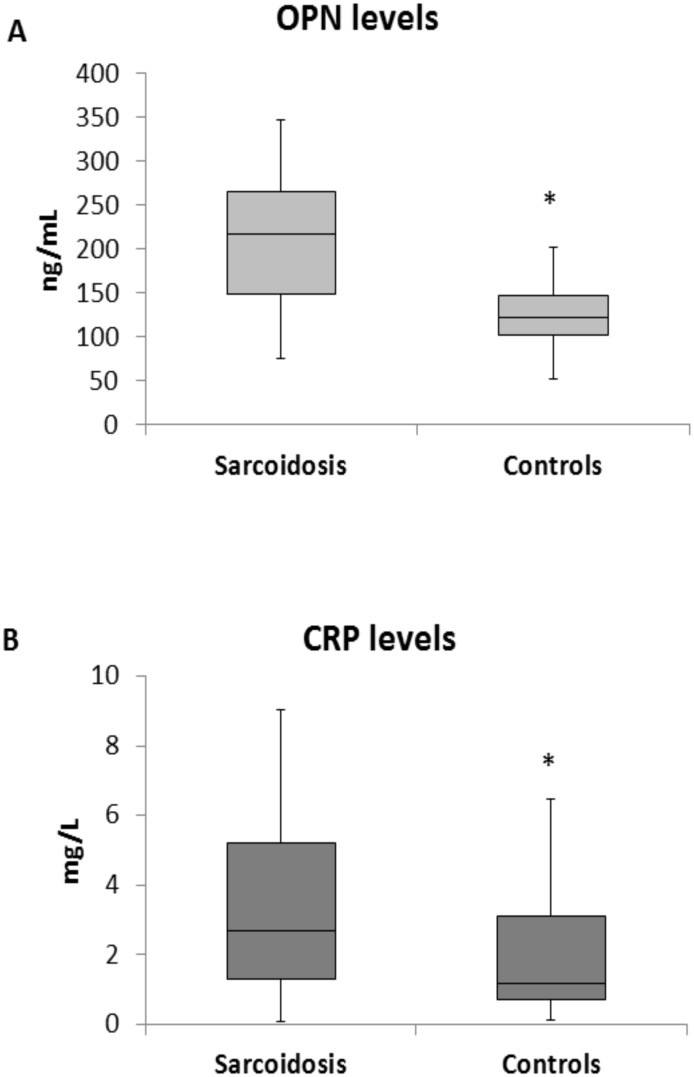
Osteopontin and CRP levels. Sarcoidosis patients had significantly higher levels of (A) plasma OPN (p<0.001) and (B) C—reactive protein (p<0.018) in comparison with healthy control subjects. Solid horizontal line indicates median. Color box indicates interquartile range. Black line indicates range of measurements. **OPN-** Osteopontin, **CRP-** c-reactive protein.

**Fig 2:**
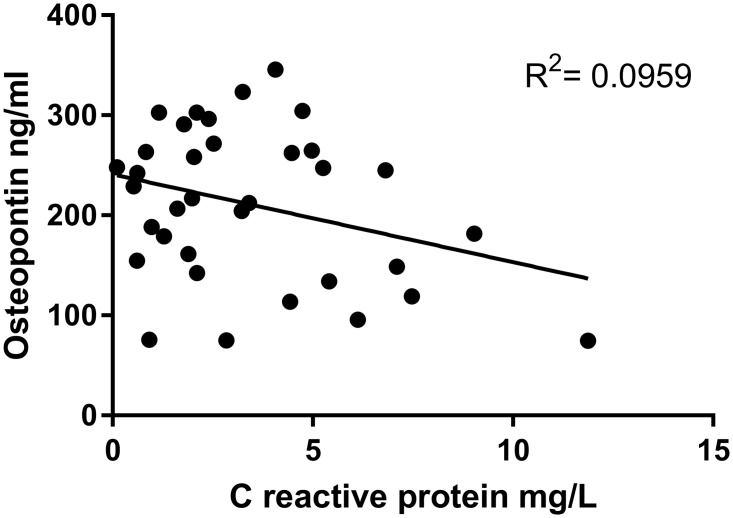
Correlation between osteopontin and C reactive protein. Plasma osteopontin level is not correlated with C—reactive protein level.

Protein levels of OPN and CRP in plasma of sarcoidosis patients and controls are presented in Fig 1. Sarcoidosis patients had significantly higher levels of OPN (patients median 217 ng/ml, 25–75% range: 149–265 vs controls median 122ng/ml, range 102–147; p<0.001), as well as CRP (patients median 2.7mg/L, 25–75% range 1.3–5.2 vs controls median 1.2mg/L, range: 0.7–3.1; p = 0.018) in comparison with healthy control subjects. We found no correlation between OPN and CRP levels (Fig 2).

We evaluated possible associations between plasma OPN levels and clinical and epidemiological parameters of disease in sarcoidosis patients. OPN levels were significantly elevated in smoking patients (p = 0.049), median levels of 247ng/ml in present and past smokers vs. 179 ng/ml in never-smokers. OPN levels were significantly decreased in patients whose presenting symptoms were rash or skin involvement (medians: 114ng/ml vs 218ng/ml, p = 0.025).

**Fig 3:**
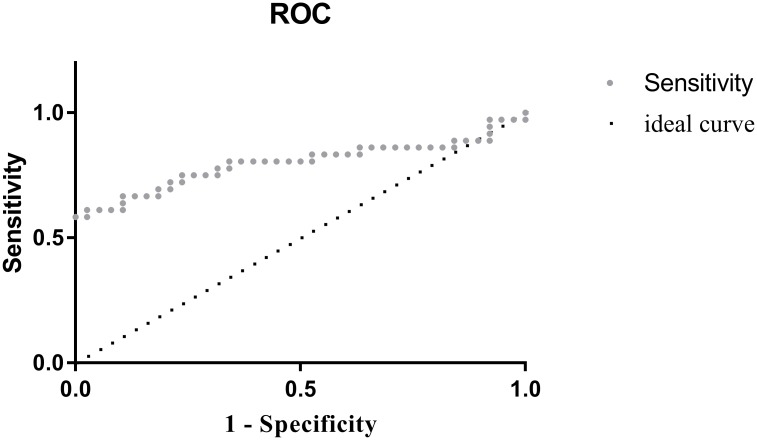
Receiver Operating Characteristic (ROC). Curve showing specificity and sensitivity percentages of osteopontin in patients and controls. Area under the curve is 0.798 (0.686–0.909 95% CI). 89.5% specificity and 63.9% sensitivity for cutoff value of 180ng/ml. Positive (sarcoid), Negative (control).

Serum OPN may serve as a diagnostic test for sarcoidosis. Using ROC curves the optimal cut-off value to differentiate sarcoidosis from healthy controls is 180ng/ml which gives a specificity of 89.5% and sensitivity of 64% (Fig 3).

### Single nucleotide polymorphisms

**Fig 4:**
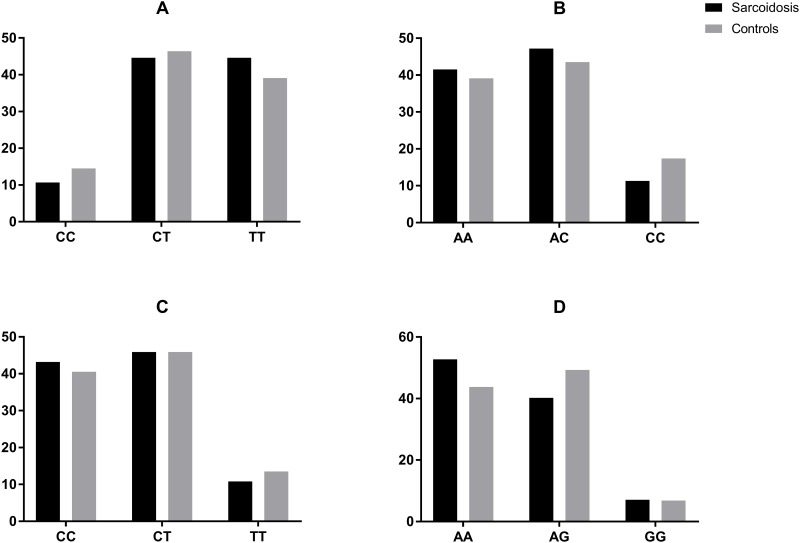
Frequency of SNPs. Frequency of SNPs in patients with sarcoidosis (clear) and controls (black) for the four allelic sites examined: each pair of columns represents percentage a possible variant of total group. (A) rs4754C/T (B) rs9138 A/C (C) rs11276616 C/T (D) rs1126772 A/G No differences were observed between groups.

We found no differences between sarcoidosis patients and controls in the frequency of the three variations in any of the four SNPs we examined (Fig 4A–4D, Table 3). We did find some significant differences in the frequency of SNP in certain patient subgroups. Parenchymal lung involvement was found related to SNP rs1126772 (p = 0.02). Presenting symptoms of fever or significant weight loss was related to SNPs at three sites: SNP rs4754 (p = 0.01); SNP rs9138 (p = 0.027); and SNP rs11276616 (p = 0.027). Plasma OPN levels were not significantly different in any variant of any SNPs for the entire group or for only the patient group.

**Table 3:** SNP frequency.

rs1126772		Patients % (n)	Controls %(n)	
	AA	52.7 (59)	43.8 (32)	49.2 (91)
	AG	40.2 (45)	49.3 (36)	43.8 (81)
	GG	7.1 (8)	6.8 (5)	7.0 (13)
Total		(112)	(73)	(185)
rs4754				
	CC	10.7 (12)	14.5 (10)	12.2 (22)
	CT	44.6 (50)	46.4(32)	45.3 (82)
	TT	44.6 (50)	39.1 (27)	42.5 (77)
Total		(112)	(69)	(181)
rs11276616				
	CC	43.2 (48)	40.5 (30)	42.2 (78)
	CT	45.9 (51)	45.9 (34)	45.9 (85)
	TT	10.8 (12)	13.5 (10)	11.9 (22)
Total		(111)	(74)	(185)
rs9138				
	AA	41.5 (44)	39.1 (27)	40.6 (71)
	AC	47.2 (50)	43.5 (30)	45.7 (80)
	CC	11.3 (12)	17.4 (12)	13.7 (24)
Total	Count	(106)	(69)	(175)

All individual data are available in S1 Table.

## Discussion

In this study, we found elevated levels of plasma osteopontin (OPN) in patients with sarcoidosis when compared to healthy controls. We found no correlation between the presence of four single nucleotide polymorphisms (SNPs) tested and plasma OPN levels or presence of disease. However, some of the clinical parameters were found to correlate with genotype.

OPN is expressed in T- cells as an early response molecule in bacterial infections. It interacts with macrophages and induces a type-1 cytokine inflammatory response, potentiating production of IL-12 and IFN- γ. OPN has also been previously linked to “Th1-diseases” such as multiple sclerosis [44], rheumatoid arthritis [21], autoimmune and viral hepatitis [45], and to other granulomatous disorders such as Vogt–Koyanagi–Harada disease (VKH). OPN has also been found to be associated with lung diseases such as idiopathic pulmonary fibrosis (IPF) and is overexpressed in bronchoalveolar lavage of IPF patients [46]. Elevated serum OPN has been described previously in sarcoidosis patients and was also expressed in granulomas of patients with sarcoidosis [24,32]. In light of the above data, it is to be expected that an association between OPN levels and the presence of sarcoidosis will be found.

We considered whether plasma osteopontin could be of use as a biomarker to diagnose sarcoidosis or as a marker of disease activity and of response to treatment. As regards diagnosis, receiver operator curves for OPN show a diagnostic value (area under the curve–AUC) of 0.798, which is similar or slightly better than existing markers, which are being using routinely. For example, angiotensin converting enzyme (ACE), which is known to be non-specific and increased in neoplastic and chronic infectious conditions, was found to have AUC of 0.779 (0.668–0.891 confidence interval) and sIL-2R had an AUC value of 0.667 (0.539–0.795 confidence interval) [47]. Chitotriosidase has recently been reported to have an AUC of 0.98 [48]. Although OPN levels may differentiate between sarcoidosis and healthy controls, we do not know whether OPN levels would be of value to differentiate between sarcoidosis and other interstitial diseases or other diseases presenting with lymphadenopathy, particularly lymphoma, but further studies are required to clarify this matter. There may also be additional modifying factors that influence levels of OPN such as treatment with inhaled corticosteroids and smoking. The optimal value for OPN to diagnose sarcoidosis depends on the pretest probability for this diagnosis. Based on our study data, a low cutoff value of 100ng/ml will provide specificity of 21%, and sensitivity 86%. When trying to rule out sarcoidosis, a higher cutoff of 223 ng/ml is preferable, with a 100% specificity and sensitivity of 47%. Optimal cutoff value of 180ng/ml gives specificity of 89.5% and sensitivity of 64%.

Our findings do not support the use of OPN for disease monitoring. We found no correlation between OPN and CRP levels in sarcoidosis patients, despite both being elevated relative to levels found in the control group. CRP is generally considered a marker of disease activity in inflammatory diseases and OPN clearly does not simply reflect the information provided by CRP. Further studies using serial measurements pre and post treatment may clarify whether there is any correlation between OPN and disease activity.

We chose to study four of OPN's SNPs based on previously published studies that found association between these specific SNPs and different autoimmune and Th1 related diseases, such as Systemic Lupus erythematosus, type I Diabetes Mellitus and rheumatoid arthritis, or Behcet`s Disease and Vogt-Koyanagi-Harada Disease, which are also characterized by aberrant Th1 and Th17 response. The alleles distribution of rs9138, rs7687316, together with allelesrs1126616T and rs1126772A have all been associated with SLE [34,35]. A significantly increased frequency of the OPN rs4754 TT genotype was observed in VKH patients compared with healthy controls [37]. In the other hand, SNPs rs1126616, rs1126772 and rs9138 were found to be related to asthma diagnosis and clinical parameters in Puerto Rican population [36], with asthma being a Th2 disease model.

As regards studies in sarcoidosis, in the Slovenian population rs4754 was found to have different genotype frequency in sarcoidosis patients, with CC being overrepresented. This was found significant, but not after correction for multiple testing. Haplotype TT for rs11730582-C/T, rs11728697-C/T and rs4754-C/T were significantly decreased and therefore considered protective [40]. In contrast to these studies, studies in patients with Behçet's disease and with type 1 diabetes showed elevated OPN levels, but failed to show connection to SNP rs1126772 [49,50]. This is similar to what we found in our study.

The failure to identify correlation between genotype and the presence of sarcoidosis in our cohort was surprising and somewhat disappointing. Although unlikely, this may be due to a type II error related to the size of our patient cohort. Another possible explanation is that we did not check for haplotypes, but for isolated SNPs, and the only correlation previously found between the genotype of OPN and the presence of sarcoidosis, after correction for multiple testing, was for the whole haplotype. The lack of correlation could also be reflecting perhaps another, non-genetic, epigenetic or other mechanism in which OPN contributes to Th1 related diseases, in a way similar to previous studies in Behçet's disease and type-1 diabetes. Lastly, correlation studies regarding SNP's are invariably population related and therefore may be different in our cohort of Israeli patients, especially bearing in mind that our patients have heterogeneous ethnic origins.

Some clinical parameters were found to correlate with the frequency of SNP's we have examined, with the presence of constitutional symptoms showing the strongest correlation. These findings could be explained in numerous ways. They could be reflecting a subset of sarcoidosis with genetic predisposition which is affected by the OPN genotype and which manifests with a prominent systemic reaction rather than as disease limited to the chest. In patients presenting with fever or weight loss, all four SNPs examined were found to have the same distribution, suggesting a similar haplotype for this presentation.

From our study it is not clear whether OPN is involved in the pathogenesis of sarcoidosis or merely a marker of the disease. If OPN is found to be relevant to the pathogenesis, it may serve as a target for future therapy. There is increasing interest in OPN and its ligands as a therapeutic target in multiple sclerosis [51], and in various cancers [52].

## Conclusion

In conclusion, our study demonstrates that there is an association between the protein osteopontin and sarcoidosis. Plasma osteopontin may be a suitable marker for the presence of sarcoidosis, but control groups with other lung diseases need to be studied. Surprisingly, no genetic association was found between osteopontin and the presence of sarcoidosis, however, a genetic association was found with some clinical subtypes of disease.

## Supporting information

S1 Table Individual data. (XLSX)
